# The Italian consensus conference on psychological therapies for anxiety and depressive disorders: findings and recommendations

**DOI:** 10.1017/S2045796022000713

**Published:** 2022-12-13

**Authors:** Angelo Barbato, Gioia Bottesi, Massimo Biondi, Mariangela Corbo, Giovanni de Girolamo, Gerardo Favaretto, Silvio Garattini, Paolo Migone, Paolo Moderato, Emiliano Monzani, Franco Veltro, Ezio Sanavio

**Affiliations:** 1 IRCCS Istituto di Ricerche Farmacologiche Mario Negri, Milano, Italy; 2 Department of General Psychology, Università degli Studi di Padova, Padova, Italy; 3 Department of Human Neurosciences, Sapienza Università di Roma, Roma, Italy; 4 Department of Mental Health, ASREM, Campobasso, Italy; 5 IRCCS Fatebenefratelli, Brescia, Italy; 6 Azienda ULSS n.2, Treviso, Italy; 7 Editor, Psicoterapia e Scienze Umane, Parma, Italy; 8 Department of Marketing, Behavior, Communication ‘G.P. Fabris’, Università IULM, Milano, Italy; 9 ASST di Bergamo Ovest, Bergamo, Italy

**Keywords:** Common mental disorders, community mental health, evidence-based psychiatry, psychotherapy

## Abstract

A Consensus Conference of clinicians, researchers, public health specialists and users was convened in Italy to review efficacy, effectiveness, treatment appropriateness and access to care for anxiety and depression, and to consider the role of psychological therapies. Expert opinion was sought concerning identification of people requiring psychological therapies according to levels of symptom severity matched to corresponding levels of treatment intensity, suitability of psychological therapies for subclinical anxiety or depression, definition of a minimum level of information on evidence-based psychotherapies to be provided by university medical and psychology courses, initiatives to raise awareness among potential users and decision makers on the role and effectiveness of psychological therapies in healthcare. The expert jury concluded that a number of psychological therapy models endorsed by most authoritative guidelines are supported by research showing their effectiveness at least equal to the drugs used in common mental disorders (CMDs). Such therapies are under-represented in the Italian public health system, leading many people to resort to the private sector, resulting in unacceptable wealth discrimination. The difficulty of accessing psychological treatments often entails the use of drug therapies in cases where they are not indicated. Starting from these assumptions, the experts recommended the promotion of better and timely recognition of anxiety and depressive disorders and their classification in terms of symptom intensity and functional impairment, differentiating subthreshold mood swings from clinical forms, to foster outcome studies of psychotherapies in CMDs in Italy, to introduce a stepped care model structured according to levels of intensity of treatment, based on wellbeing support strategies in nonmedical contexts for subthreshold situations, self-help, support and psychoeducation as frontline interventions in mild clinical forms, evidence-based psychotherapies in moderate and severe forms, with the option of combining psychological treatment and appropriate drug therapy in the most severe cases.

## Psychological therapies for anxiety and depressive disorders: where do we stand?

Anxiety and depression are common mental disorders (CMDs). In the Italian website of the *World Mental Health Survey Initiative* (de Girolamo *et al*., 2006), a representative random sample of non-institutionalised Italian citizens aged 18 or older (*N* = 4712) was interviewed using Version 3.0 of the *Composite International Diagnostic Interview* (CIDI): 11% of respondents reported a lifetime history of some sort of mood disorder (excluding bipolar disorders) and 10.3% of anxiety disorder. Antidepressant drugs and psychotherapies are considered the mainstay of evidence-based treatment of depression by all guidelines. Both drugs and psychotherapies are effective in reducing depressive symptoms, although high placebo response rates of around 30% are well documented and effect sizes achieved by both treatments in comparison with placebo or usual care are quite small, clustering around 0.30 (Cuijpers *et al*., 2014; Jakobsen *et al*., 2017; Leichsenring *et al*., 2022). When directly compared, psychological and pharmacological treatments do not show any short-term differences (Cuijpers *et al*., 2013). Small, clinically not relevant, differences have been found among different antidepressant drugs (Jakobsen *et al*., 2017) and different types of psychological interventions (Cuijpers *et al*., 2021), including cognitive-behavioural, psychodynamic, problem-solving and interpersonal approaches. Clear-cut evidence of efficacy for any treatment is limited to depression with severe symptoms, when antidepressant medications are often considered the mainstay of treatment (Weitz *et al*., 2015). Some data suggest that combination of psychotherapy and drug therapy might be better than both treatments alone (Cuijpers *et al*., 2020). The available data do not support the use of drug therapy in mild and subthreshold depression, in which low intensity psychosocial support, as well as a number of non-conventional treatments (such as intensive physical activity) have proved to be effective (Gartlehner *et al*., 2016; Klein *et al*., 2016; Pearce *et al*., 2022). Some evidence also suggests that psychological treatments might be more effective than drugs in preventing depression relapse or recurrence in the long term (Bockting *et al*., 2015).

Overall, the previous considerations are valid for anxiety disorders (Carl *et al*., 2020), especially considering that CMDs are experienced as dimensional phenomena and comorbid anxious and depressive problems are usual in clinical practice. People with mental disorders consistently report that talking therapies are preferred over drugs (McHugh *et al*., 2013).

Complaints related to anxiety and depressive symptoms are an important driver for help-seeking behaviour, and primary care should be a key setting for their treatment, since they rank among the most common problems presented by primary care adult patients in Italy (Mazzoleni *et al*., 2011). However, when people with CMDs seek help, their distress is often recognised only when moderate or severe; in any case, the treatment is mostly focused on antidepressant and/or anti-anxiety drugs (Balestrieri *et al*., 2004), even in people with mild depression (Demyttenaere *et al*., 2008). Prescriptions of antidepressant drugs in Italy have been rising steadily in the past twenty years, reaching 44.6 DDD/1000 population in 2021 (Osservatorio Nazionale sull'Impiego dei Medicinali, 2022). There are indications of inappropriate antidepressant use (Lunghi *et al*., 2020).

Some international studies also showed a reduction of social and health costs in samples of individuals with depression treated with psychotherapy (Fournier *et al*., 2015; Gajic-Veljanoski *et al*., 2018). Even a modest increase (10%) in recovery rates would cover the costs of increased access to psychological therapies, as occurred in Britain: *Improving Access to Psychological Therapies* (IAPT) programme (Clark, 2018).

## Mental health care and psychotherapy in Italy

Italy has been widely known internationally in the area of mental health for the ‘Law 180’ approved in 1978, which brought about radical changes in mental health care. Italy's ‘Law 180’ approved in 1978 and widely recognised in international mental health circles, brought about radical changes in mental health care. The 80 mental hospitals active in 1978 were shut down in only a few years, and a comprehensive system of mental health services was developed: in 2020 (last official data) there were 134 Departments of Mental Health providing care to 728 338 citizens suffering from different types of mental disorders (Ministero della Salute, 2021). In contradiction to the radical orientation of Law 180, no provision was made for the training of professionals who carry the main burden of responsibility of implementing the general principles underlying the reform. Neither residency programmes for psychiatrists nor university training for clinical psychologists has changed significantly over the years, and this has a marked impact on the quality of mental health care, as shown by several studies published in the last four decades (Volpe *et al*., 2014). In addition, several surveys found that psychosocial treatments, including formal psychotherapies, are not widely available and are underused for the treatment of patients who may be in need of them (Barbato *et al*., 2016).

In 1989 a new law established a number of conditions which have to be met to be licensed as a psychotherapist. It established that professionals aiming to be licensed have to attend a 4-year postgraduate school (paid privately, since publicly funded university schools are very rare) recognised by the Ministry of University and Research (MIUR).

The same law established that the residency training programmes in Psychiatry and Child and Adolescent Psychiatry also automatically lead to the license of ‘psychotherapist’.

There are currently 389 official schools providing training in psychotherapy; the criteria for the recognition of these schools, for the content of the teaching syllabus, and for the qualification of teachers are mostly formal (e.g. number of hours of teaching). These schools embrace all kinds of ill-defined theoretical orientations, without regard to the evidence base of research on psychotherapy models (Migone, 2020).

According to the Italian Board of Psychology, in 2020 in Italy there were as many as 117 762 psychologists (Consiglio Nazionale Ordine Psicologi, www.psy.it/dati-statistici). It is estimated that there are more than 50 000 licensed psychotherapists; most of them work in private practice, which makes it extremely difficult to access reliable data about their clinical activities, in terms of number of people in treatment, prevailing diagnostic profiles, treatment outcomes, etc. For these practitioners further education is not formally required, although both psychiatrists and psychologists are required to obtain annual CME credits for attending relevant courses.

While there are regulatory agencies (e.g. EMA, FDA, AIFA) controlling the availability of medications and medical devices as well as the conditions for their use, regulatory agencies active in the area of psychotherapy do not exist. This is a critical issue, since the practice of psychotherapy falls under the same legal obligations (e.g. imprudence, inexperience and negligence) as any interventions targeted at people suffering from physical or mental disorders.

## The development of the Consensus Conference

Such a complex scenario gave rise to the idea of a Consensus Conference at the end of the Congress entitled ‘*Psychological therapies for anxiety and depression: Costs and benefits*’, held in Padua in 2016. Participants approved a call for convening a National Consensus group to review extant psychological treatments for anxiety and depression and promote a set of recommendations aimed at improving training, availability, and delivery of psychological treatments for these disorders (Research Group for Treatment for Anxiety and Depression, 2017). A group of Italian psychiatrists and psychologists responded to this invitation and set up the Sponsoring Committee of this Consensus Conference, led by Ezio Sanavio, Professor of Clinical Psychology at the University of Padua.

The Sponsoring Committee started work in 2018 and appointed a Scientific Committee. The aims of the Scientific Committee were as follows: to gather evidence about efficacy and effectiveness of psychological therapies for anxiety and depression; to review their patterns of use in Italy; to identify the best ways to disseminate information about evidence-based treatments; and to make them accessible within the Italian National Health Service (NHS). The Committee singled out a multidisciplinary group of experts, belonging to the fields of psychology, psychiatry, social work, general medicine, professional associations and members of users' associations. They were asked to answer a set of questions covering four broad areas: professional skills and training; efficacy and cost-effectiveness; organisational models; dissemination and information. To perform this task, the experts formed four groups drafting several background papers, subsequently merged into a final document submitted by the Sponsoring Committee to an independent jury. This was requested to formulate recommendations based on a scrutiny of the overall report. The jury was made up of 19 members, including experts in health sciences, communication, education, law and social security. A total of 61 persons, covering different professional roles, participated in the work of the Conference over more than three years, from 2018 to 2021 (Sanavio, 2022). The full list of participants, the proceedings and the background papers are available on the website of the Italian National Institute of Health (*Istituto Superiore di Sanità*) (www.iss.it/documents/20126/0/Consensus_1_2022_EN.pdf).

## Questions for the experts

The experts initially reviewed data about efficacy, effectiveness, appropriateness of treatment and access to care for anxiety and depression using international guidelines, paying special attention to the strategies aiming to reduce the number of untreated people needing care. Two further questions addressed the identification of people requiring psychological therapies according to levels of symptom severity, matched with corresponding levels of treatment intensity, and the suitability of psychological therapies for subclinical anxiety or depression. Additional questions focused on the training of professionals, including the definition of a minimum level of information on evidence-based psychotherapies provided by medical and psychology schools, the postgraduate training necessary for physicians and psychologists to be licensed for practising psychotherapy and, finally, recommendations for continuing medical education and professional development in the area of psychotherapy.

The various committees of the Consensus Conference examined the guidelines suggested by the main scientific organisations of the USA, United Kingdom and Australia., as described in the Final Document of the Consensus Conference (Working Group ‘Consensus on Psychological Therapies for Anxiety and Depression’, 2022), and reviewed also the main meta-analyses on the treatment of CMDs (anxiety and depression). Many forms of psychotherapy for depressive and anxiety disorders are empirically based, and purposely the Final Document did not specify in detail all forms and techniques of psychotherapy for the various depressive and anxiety disorders, but only some of them otherwise the document would have resulted too long. However, this is specified later in the sections on recommendations. The members of the various committees of the Consensus Conference were representative of different orientations, such as CBT, psychodynamic therapy, etc.

## Report presented to the jury

The conditions identified as CMDs are largely heterogeneous, and the severity level plays an important role. It seems reasonable to consider anxiety and depression as dimensional phenomena with respect to which individual cases are positioned along a continuum of severity. The importance of severity assessment to improve treatment is highlighted by the discrepancy between the heterogeneity and complexity of the characteristics of people seeking help, and the prevailing pattern of pharmacotherapy in health settings. For instance, 80% or more of the cases with depression in primary care receive antidepressant drugs (Mazzoleni *et al*., 2011).

To address this problem, the Scientific Committee recommended an approach based on levels of treatment intensity (‘*stepped care*’). This requires that health professionals should not only detect anxiety and depression, but also assess their severity: to pursue this aim, widely used tools such as the *Patient Health Questionnaire-9* (PHQ-9) for depression and the *Generalized Anxiety Disorder Scale-7* (GAD-7) for anxiety, can be considered reliable and valid (Stochl *et al*., 2022). The severity assessment allows us to offer suitable interventions of varying intensity. The first level relies on guided self-help and low intensity psychosocial support, usually delivered in primary care. Upper levels imply referral to clinicians for structured individual, group or couple psychotherapies; if appropriate, they can be accompanied by other psychosocial interventions or drug treatment in specialist settings. Assessments based on levels of severity are useful when delivered in a context making use of resources allocated to psychological therapies in primary care, as happened in England with the IAPT programme (Muntingh *et al*., 2016).

Although official health policy in Italy aims at guaranteeing access and coverage of adequate treatment for mental disorders, a number of surveys show that most citizens with CMDs do not have access to psychotherapy, despite its inclusion in the core benefit package of interventions to be provided by the National Health Service (Di Cesare *et al*., 2021). Moreover, among those who do have access, only a proportion receives treatments of proven efficacy. Therefore, given the limited availability of psychotherapies in the Italian NHS, many people requiring this type of intervention are driven to private (out-of-pocket) delivery of psychotherapy.

The Scientific Committee selected three reference guidelines for anxiety disorders: *National Institute for Health and Care Excellence* (United Kingdom), 2020; *American Psychiatric Association* (USA), 2013; and *American Psychological Association* (USA), 2017. Five guidelines for depressive disorders were considered: *National Institute for Health and Care Excellence* (United Kingdom), 2021; *American Psychological Association* (USA), 2017; *American Academy of Pediatrics* (USA), 2018; *American College of Physicians* (USA), 2016; and *Orygen Youth Health Clinical Program* (Australia), 2017. The Consensus Conference report states that these guidelines apply to the Italian health and social context, although some adaptation to the specific features of Italian health services are required. Moreover, professionals need to receive adequate training in the recommended psychotherapeutic techniques.

## E-mental health and new frontiers of psychological treatments

The use of the new technologies to deliver psychological therapies was suggested as a tool to improve access to care for people with CMDs. Research on e-mental health has grown exponentially in recent years, especially due to the restrictions on face-to-face contacts during the COVID-19 pandemic. Research focusing on online assessment shows that screening and monitoring tools can be easily adapted to the online environment without threatening their reliability. With respect to treatment, most efficacy studies of psychological therapies delivered online in CMDs have produced outcomes similar to those observed for treatments delivered in person (Vis *et al*., 2018). However, as far as self-guided psychotherapy delivered via internet is concerned, some systematic reviews suggested that contradictory findings from some studies raise concerns about its benefits. Moreover, unguided internet psychotherapy shows limitations that should be addressed before it is introduced in routine care, such as dropout rates, small effects compared with face-to-face and guided internet interventions, and possible participant selection bias (Karyotaki *et al*., 2021). Some limited Italian experiences confirm the pros and cons of these interventions (Favaretto and Zanalda, 2018).

In conclusion, a number of aspects of e-mental health, such as online self-assessments, text messaging and videoconferences can now be considered as standard practice in community mental care. Online structured psychotherapy is promising but needs further real-world research before it can be disseminated in everyday practice. In this area it is necessary to establish transparent rules and appropriate training for professionals willing to provide online therapy, and ethical concerns about fairness, access, privacy and informed consent should be addressed.

## Dissemination and information

Official media (e.g. press, radio and TV) as well as social media (e.g. Facebook, blogs, etc.) have been devoting an increasing amount of space to mental health issues, and anxiety and depression, given their high prevalence, have become popular topics for media reporting and entertainment. Unfortunately, much of the information conveyed by the media is often not scientifically sound, and blurs scientific data with personal accounts, philosophical thoughts, etc., leading to severe difficulties in the proper acquisition of data, and limitations regarding any treatments.

## Some final recommendations

The jury has produced a considerable set of recommendations; space limitations prevent us from providing a full list of these, and we therefore concentrate on a limited set of points which seem worthy of consideration in any discussion of the psychotherapy of depression and anxiety. There are three key points:

1. Not all psychological treatments can be recommended. However, some of them are endorsed by the most authoritative guidelines and a considerable body of research shows that they are at least as effective as medications used in CMDs. This refers to the cognitive-behavioural, interpersonal, problem-solving and psychodynamic approaches
2. Such psychological treatments are under-represented in the Italian NHS, obliging most people to turn to the private sector. Such an issue determines unacceptable wealth discrimination in access to health care; in addition, the private sector is often an uncontrolled area, which can create problems in cases of serious disorders.
3. Given the high incidence and prevalence of CMDs, the difficulty in accessing psychological treatments often entails the consequent use of medications, even when they do not represent first-line treatment.

Based on these assumptions, the jury formulated a number of recommendations: a selection is shown in Table 1.

**Table 1.** Selection of recommendations of the ‘Italian consensus conference on psychological treatments for anxiety and depression’

Promote a better and timely recognition of anxiety and depressive disorders and a proper assessment of symptom intensity and functional impairment: the latter should guide the choice of treatment.
Introduce a stepped care model, starting from wellbeing support strategies in non-medical settings for subthreshold situations; self-help, support and psychoeducation as frontline interventions in mild clinical forms; evidence-based psychotherapies in moderate and severe forms, with the option of combining psychological treatment and appropriate pharmacotherapy in the most severe cases.
Access to the first level of stepped care in case of subclinical problems of anxiety and depression is solely recommended for people with a relevant physical or mental comorbidity, who have suffered from anxiety and depression in the past, especially if they are in a specific ‘transitional’ phase of their life, such as adolescence, old age, parenting in the perinatal period, and wherever there is a high risk of psychopathology or a serious drop in school, social and professional performance.
Monitor the outcome of interventions both in public and private settings, to improve accountability and produce data to assess user satisfaction and therapeutic alliance, trying to evaluate the differences between efficacy (in research setting) and effectiveness (in real clinical practice) of psychological therapies.
Investigate specific aspects of treatment relating to childhood and adolescence as fundamental developmental ages.
Ensure homogeneity in treatment delivery across the country.
Increase the number of state schools for the training in psychotherapy, and improve the training delivered to undergraduate students in the field of anxiety and depressive disorders.
Demand that the Technical Advisory Committee for the Psychotherapy Schools of the Italian Ministry of Universities and Research set specific goals for training and internships aimed at improving the knowledge and use of evidence-based practices.

## Conclusions

Psychological treatments for depression and anxiety play a key role in the overall listing of interventions aimed at reducing suffering and impairment in the psychosocial functioning of people with CMDs. Much has to be done in Italy to improve timely access to these treatments, to make sure that they are properly delivered, and to guarantee the accountability of the health professionals providing them.
